# Supplementary material for: VISTA Emerges as a Promising Target against Immune Evasion Mechanisms in Medulloblastoma
Source: Cancers (Basel). 2024 Jul 24;16(15):2629. doi: 10.3390/cancers16152629 (PMC11312086; doi:10.3390/cancers16152629)

Figure S1

(a) Brain TILs (Full gating- CD45 bead enrichment)

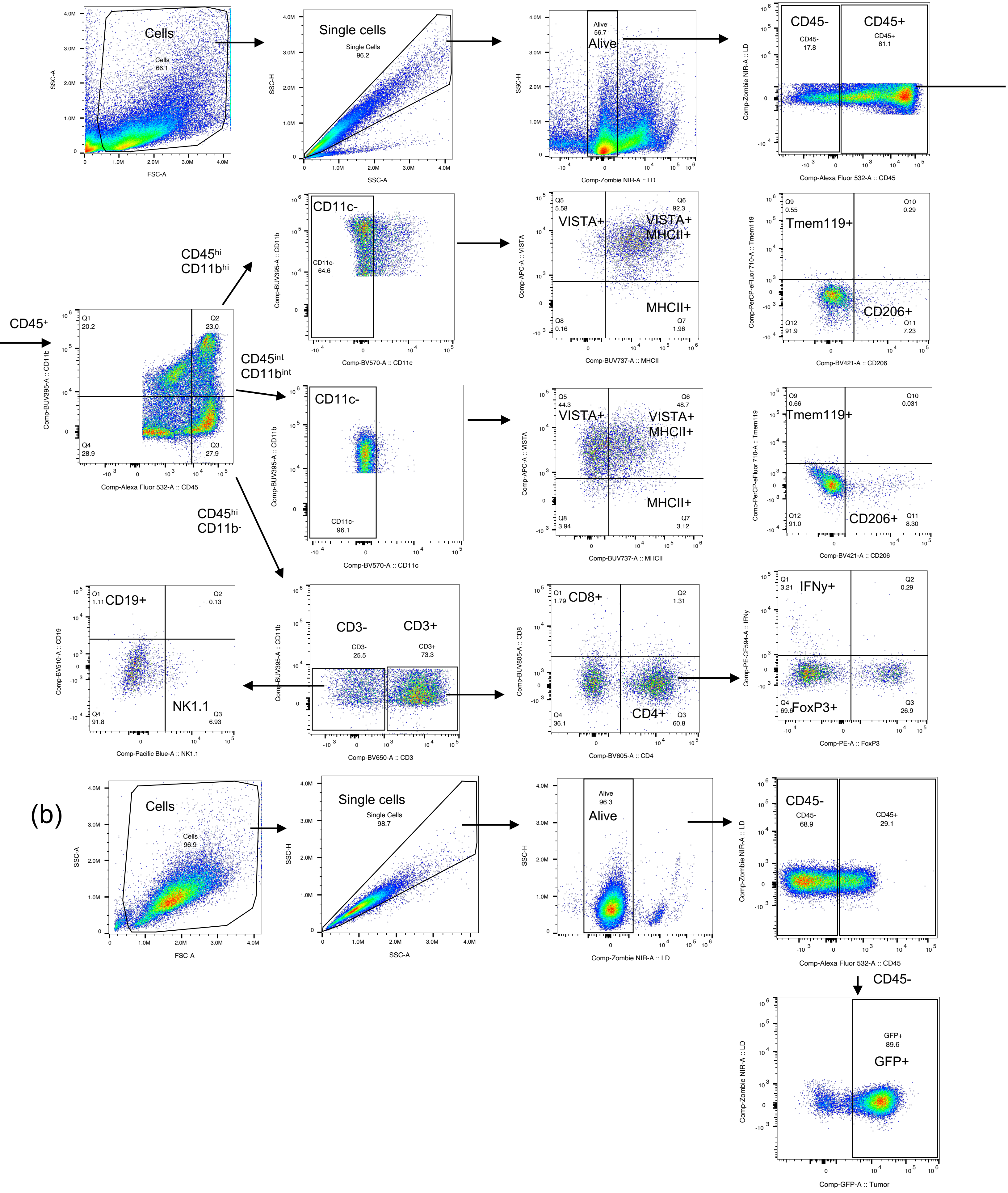

Figure S2

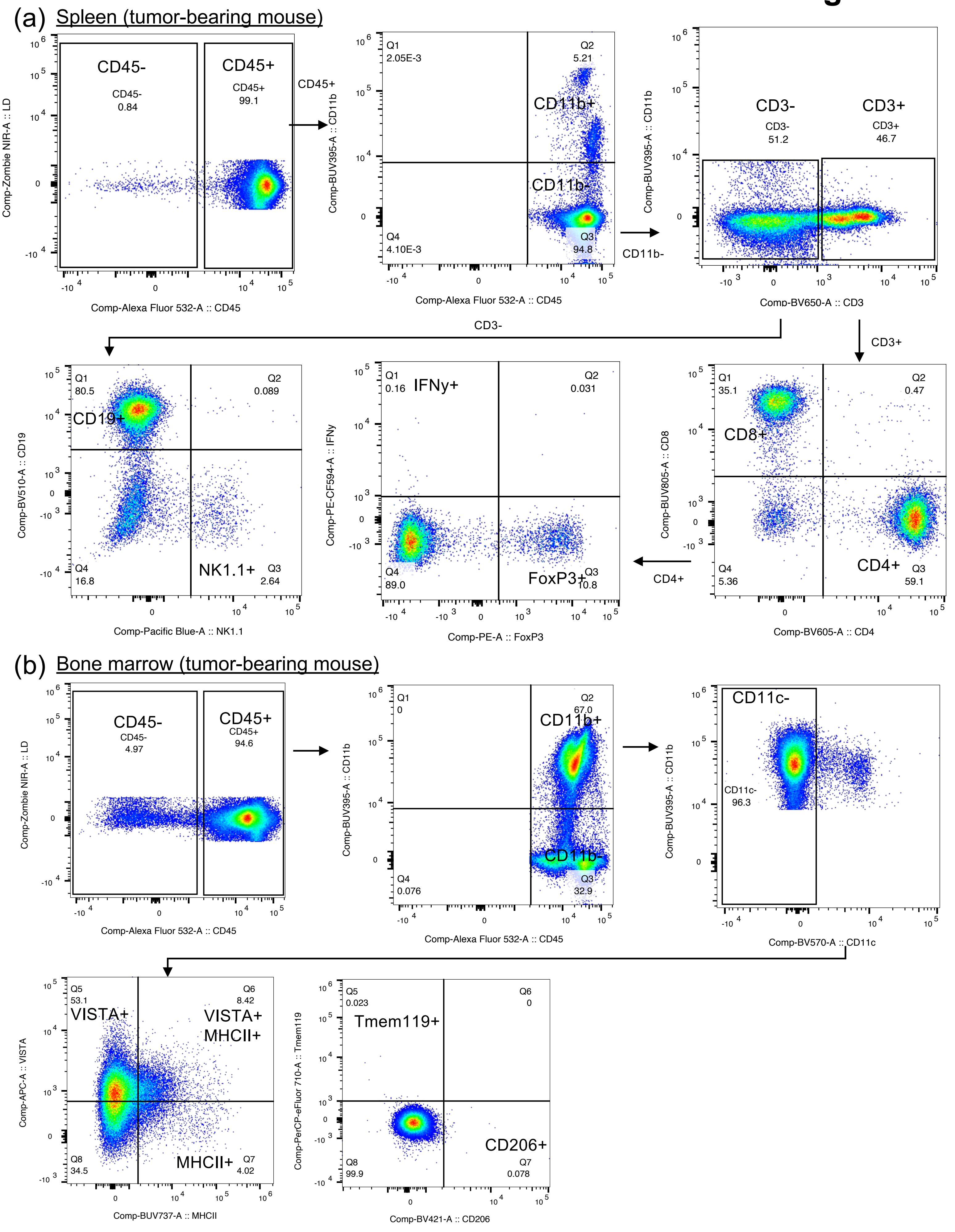

(a) Spleen (tumor-bearing mouse)
CD45-
CD45-
0.84
CD45+
CD45+
99.1
Comp-Zombie NIR-A :: LD
Comp-Alexa Fluor 532-A :: CD45
CD45+
Q1
2.05E-3
Q2
5.21
CD11b+
CD11b-
Q4
4.10E-3
Q3
94.8
Comp-BUV395-A :: CD11b
Comp-Alexa Fluor 532-A :: CD45
CD11b-
CD3-
CD3-
51.2
CD3+
CD3+
46.7
Comp-BUV395-A :: CD11b
Comp-BV650-A :: CD3
CD3-
CD3+
Q1
80.5
Q2
0.089
CD19+
Q4
16.8
NK1.1+
Q3
2.64
Comp-BV510-A :: CD19
Comp-Pacific Blue-A :: NK1.1
Q1
0.16
IFNy+
Q2
0.031
Q4
89.0
FoxP3+
Q3
10.8
Comp-PE-CF594-A :: IFNy
Comp-PE-A :: FoxP3
CD4+
Q1
35.1
Q2
0.47
CD8+
Q4
5.36
CD4+
Q3
59.1
Comp-BUV805-A :: CD8
Comp-BV605-A :: CD4
(b) Bone marrow (tumor-bearing mouse)
CD45-
CD45-
4.97
CD45+
CD45+
94.6
Comp-Zombie NIR-A :: LD
Comp-Alexa Fluor 532-A :: CD45
Q1
0
Q2
67.0
CD11b+
CD11b-
Q4
0.076
Q3
32.9
Comp-BUV395-A :: CD11b
Comp-Alexa Fluor 532-A :: CD45
CD11c-
CD11c-
96.3
Comp-BUV395-A :: CD11b
Comp-BV570-A :: CD11c
Q5
53.1
VISTA+
Q6
8.42
VISTA+
MHCII+
Q8
34.5
MHCII+
Q7
4.02
Comp-APC-A :: VISTA
Comp-BUV737-A :: MHCII
Q5
0.023
Tmem119+
Q6
0
Q8
99.9
CD206+
Q7
0.078
Comp-PerCP-eFluor 710-A :: Tmem119
Comp-BV421-A :: CD206

Figure S3

mCB MYC DNp53 (tumor-bearing mice)

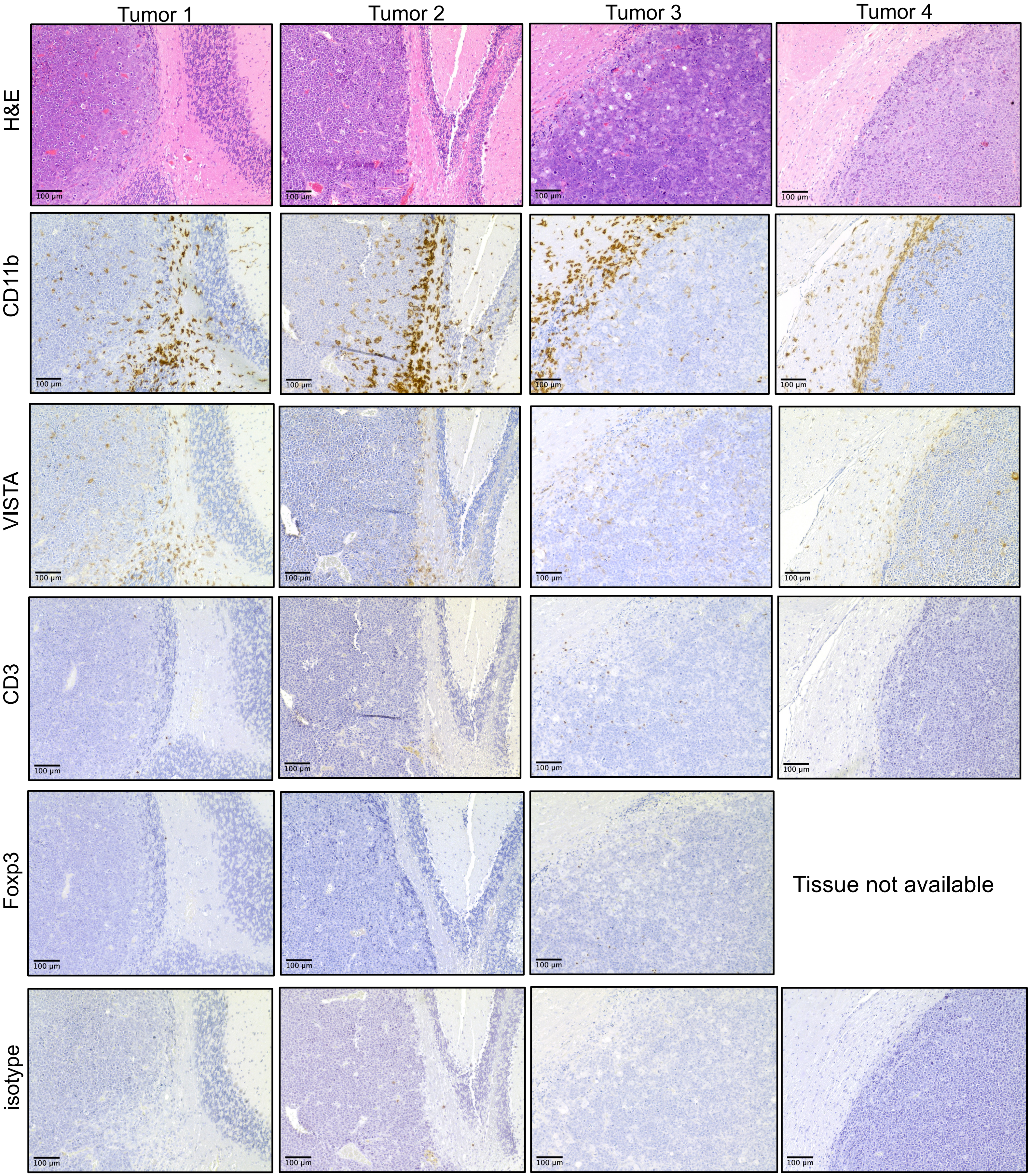

Figure S4

(a) mCB MYC DNp53 (tumor-bearing mouse)

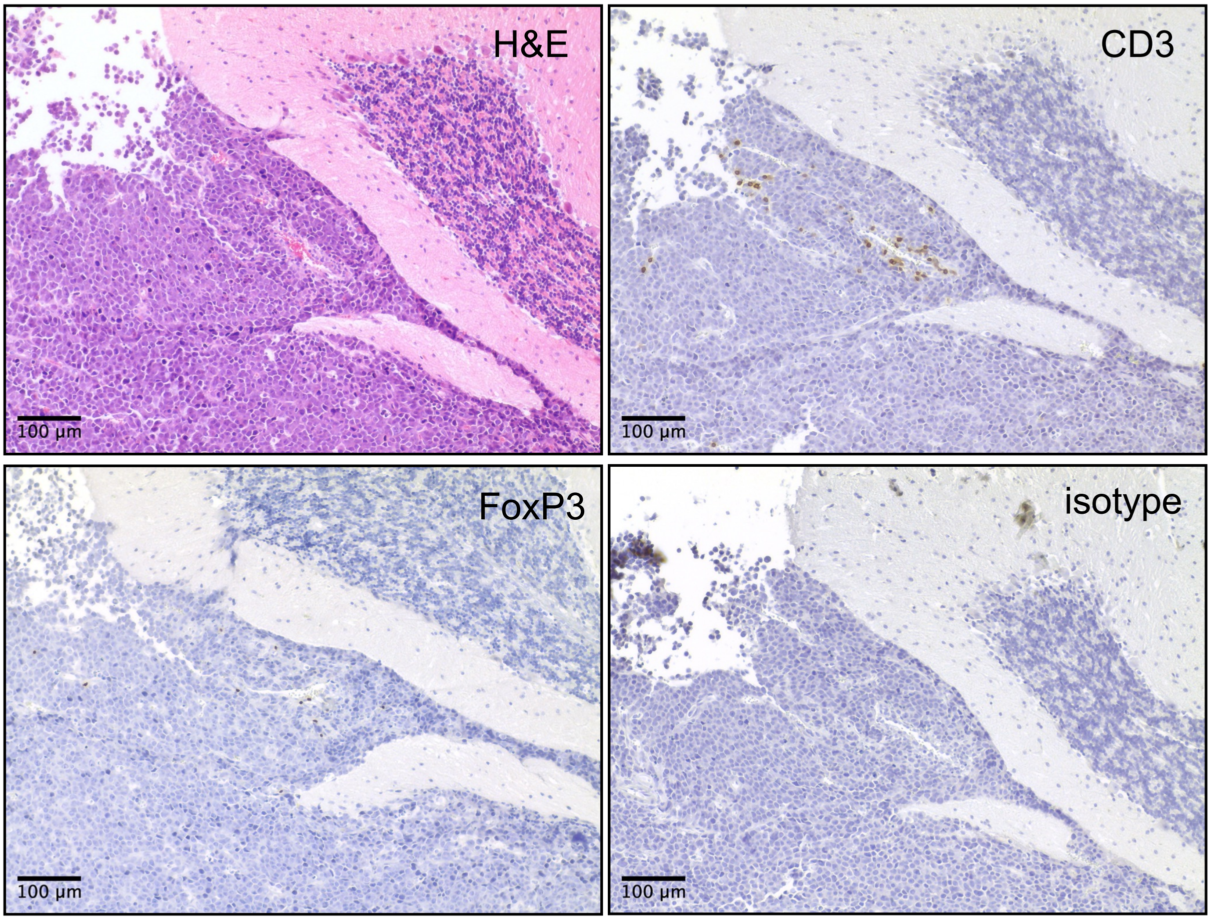

(b) Spleen (tumor-bearing mouse)

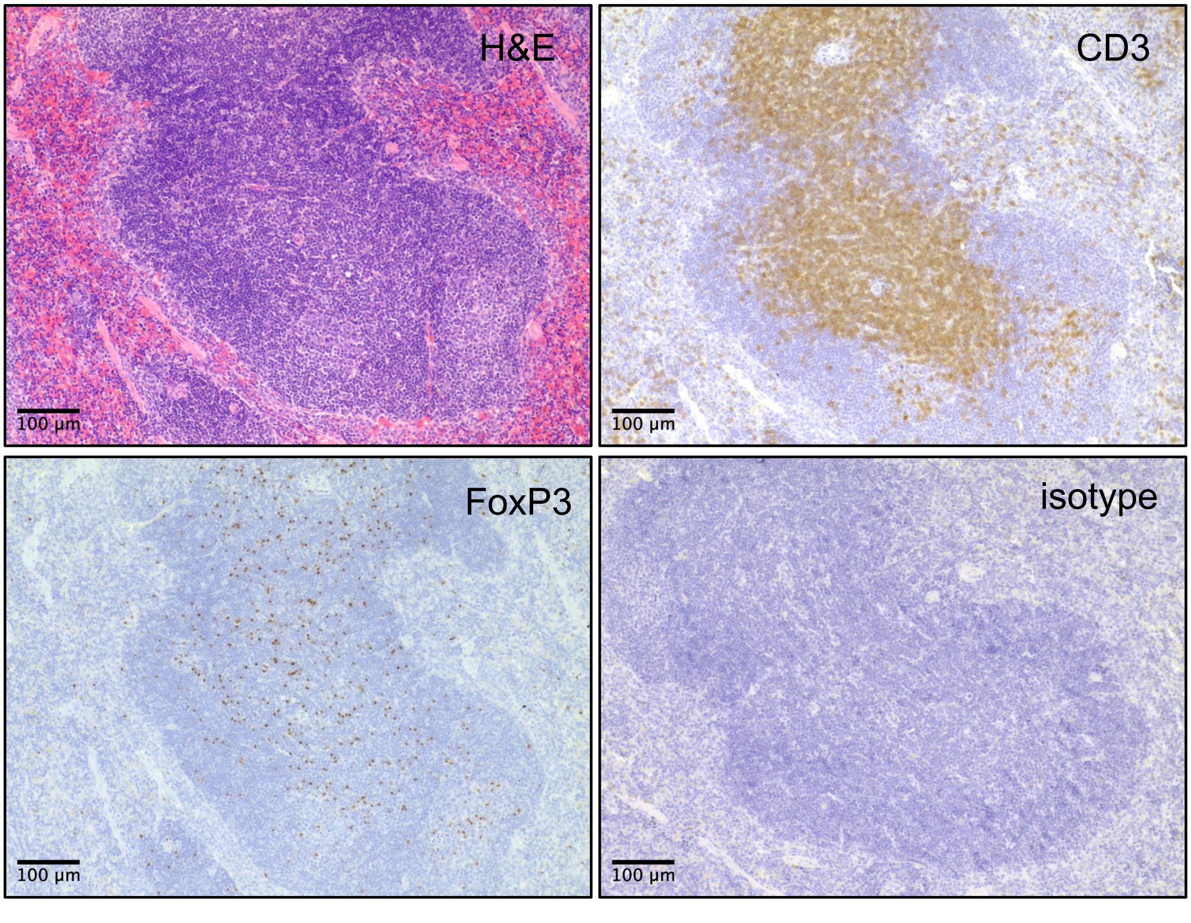

Figure S5

(a)

VSIG3			
Cell line	Sample size (n)	Average (freq of parent)	SD
D283-MED	3	71.4	34.29562654
D425-MED	1	27.1	n/a
mCB DNp53 MYC	3	86.2	15.17399091

VSIG8			
Cell line	Sample size (n)	Average (freq of parent)	SD
D283-MED	2	34.265	43.74869655
D425-MED	3	37.01	32.42954363
mCB DNp53 MYC	4	46.55	29.56692972

(b)

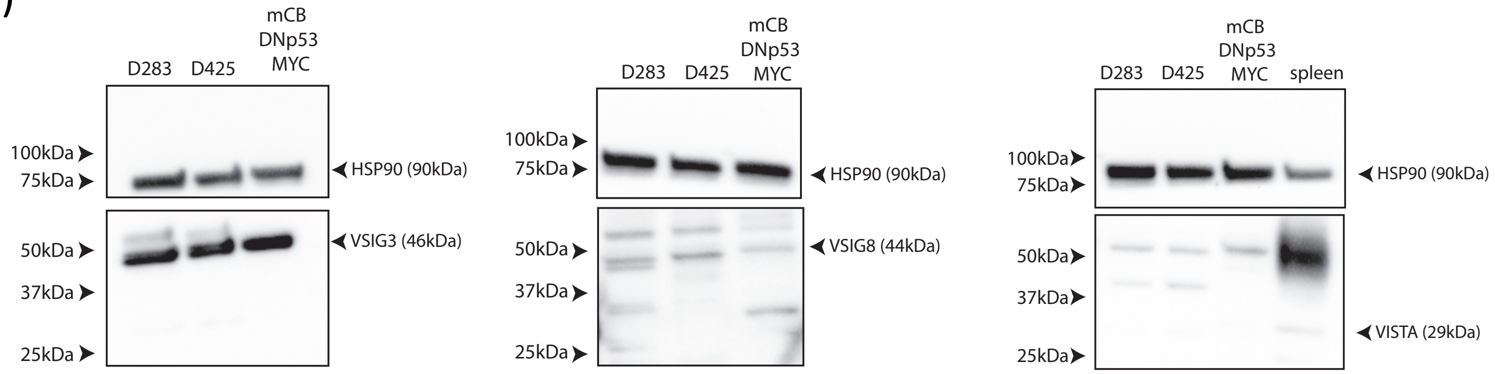

(c)

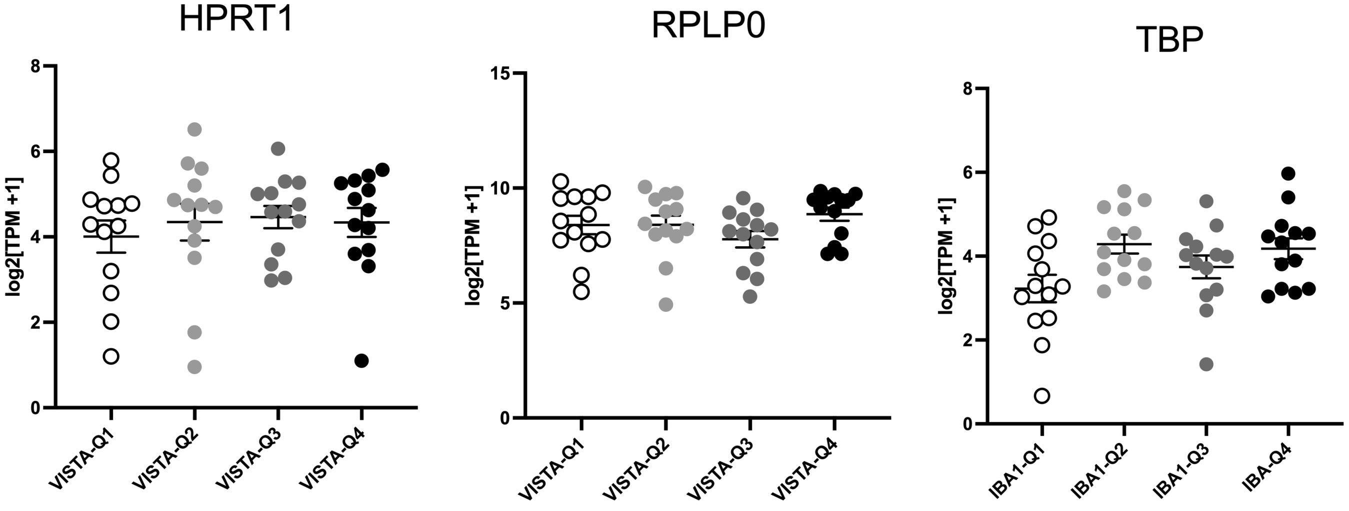

Supplement: Supplementary file 1 [file cancers-16-02629-s001.zip › File S1. figures.pdf]
