# Supplementary figures and images for: VISTA Emerges as a Promising Target against Immune Evasion Mechanisms in Medulloblastoma
Source: Cancers (Basel). 2024 Jul 24;16(15):2629. doi: 10.3390/cancers16152629 (PMC11312086; doi:10.3390/cancers16152629)

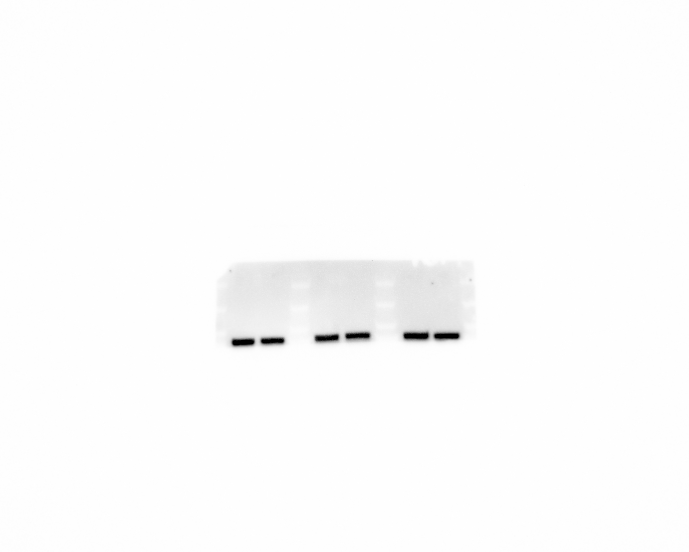

Supplement: Supplementary file 1 [file cancers-16-02629-s001.zip › File S2. Western blot/hsp90_1.tif]

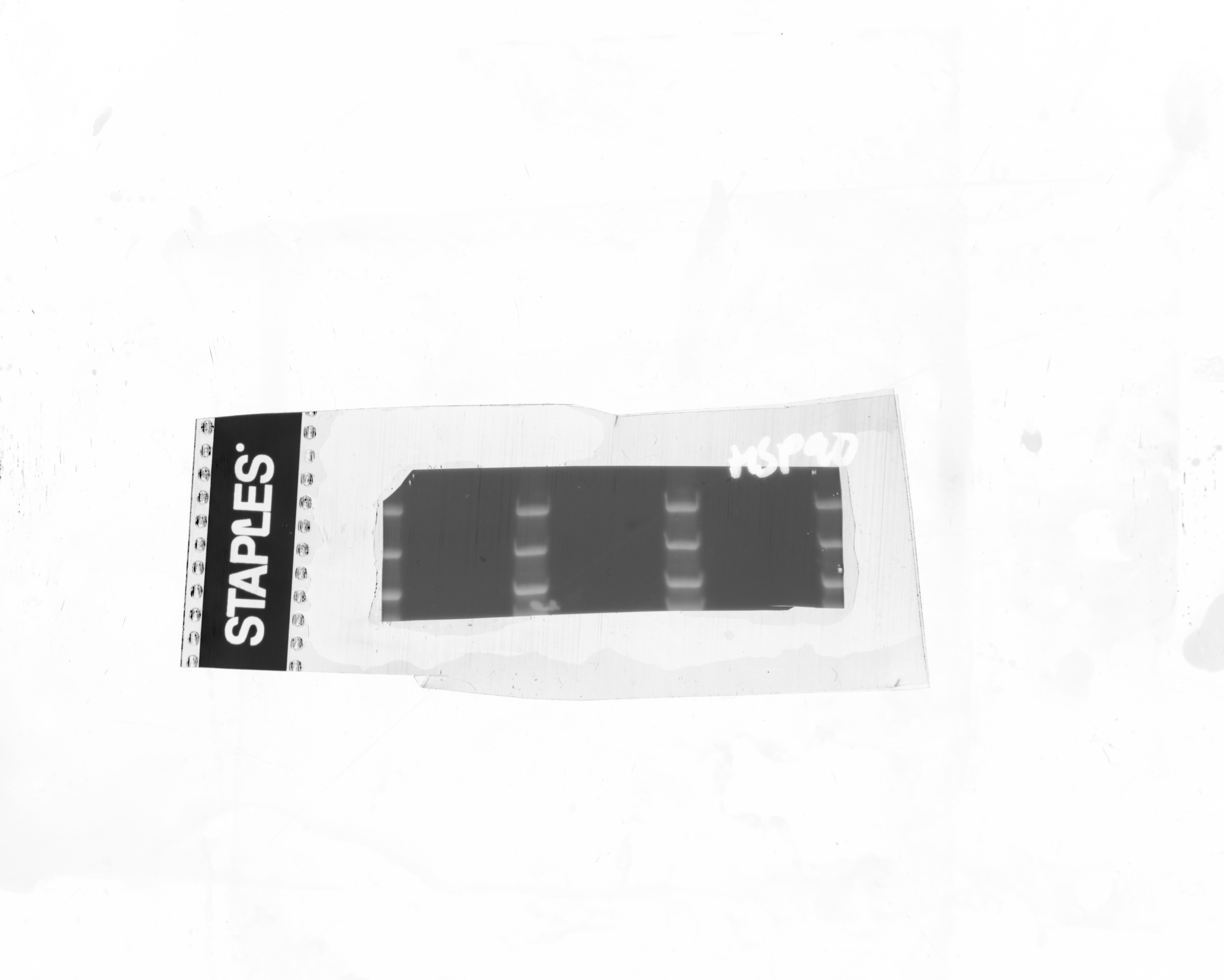

Supplement: Supplementary file 1 [file cancers-16-02629-s001.zip › File S2. Western blot/hsp90_2 ladder.tif]

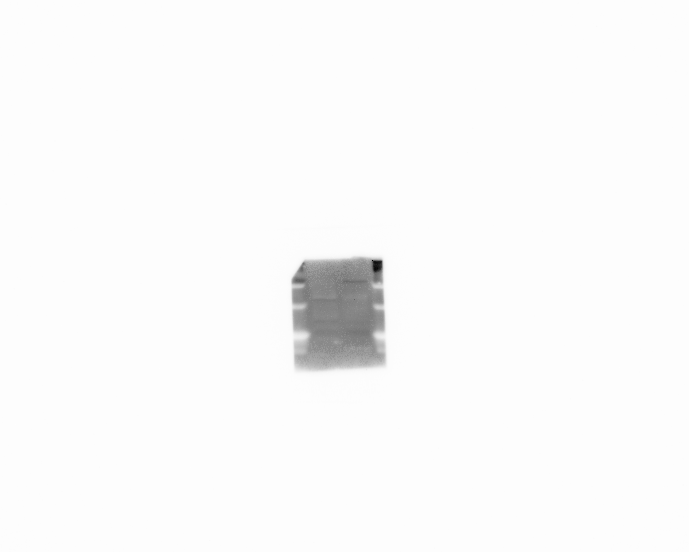

Supplement: Supplementary file 1 [file cancers-16-02629-s001.zip › File S2. Western blot/vista_1.tif]

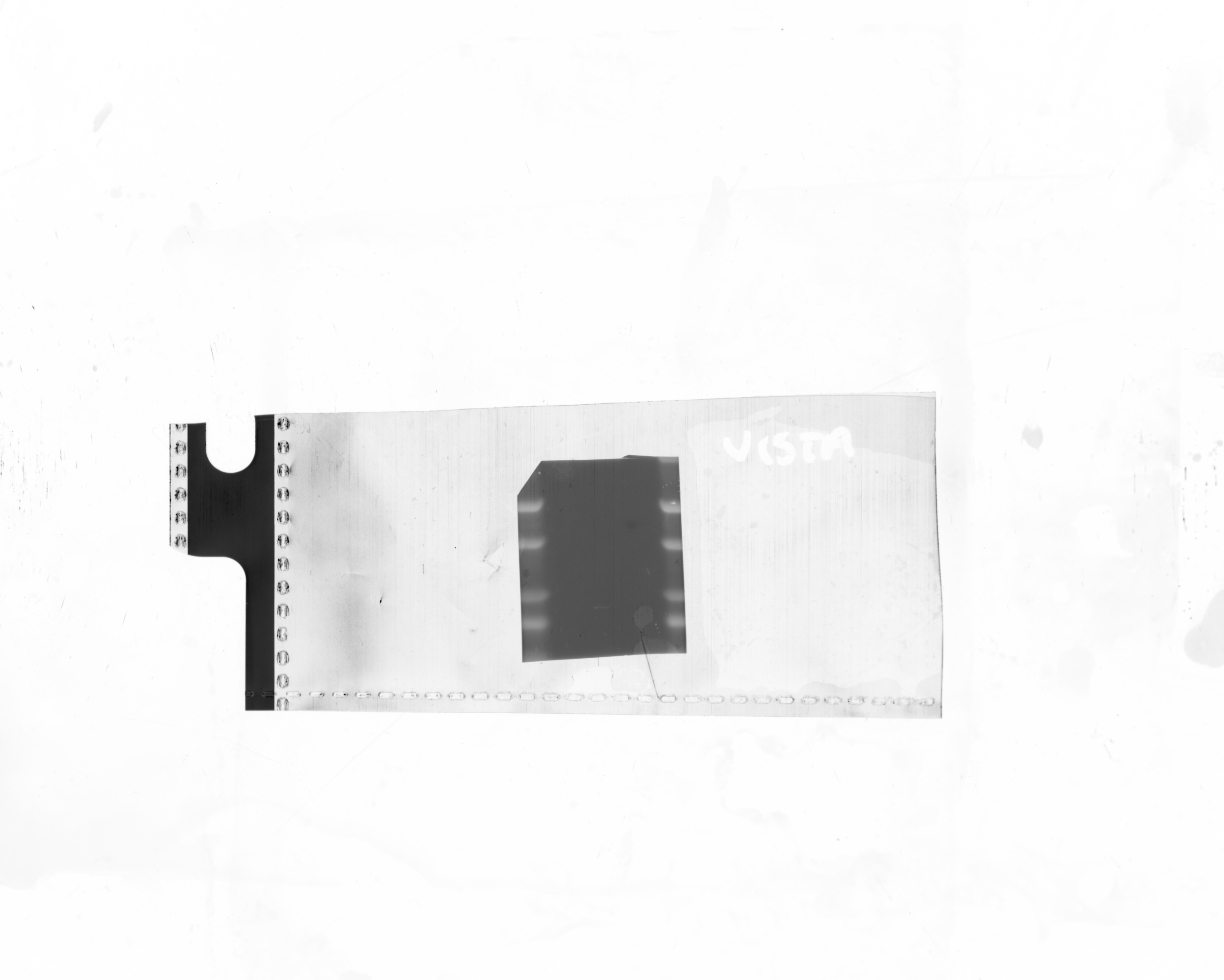

Supplement: Supplementary file 1 [file cancers-16-02629-s001.zip › File S2. Western blot/vista_2 ladder.tif]

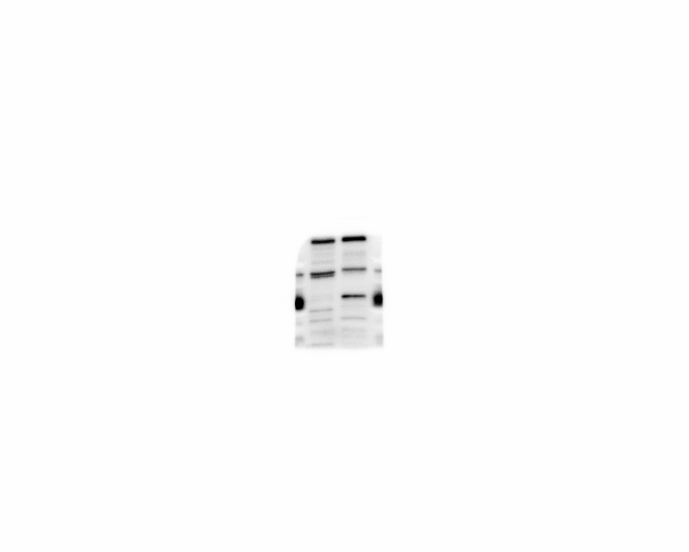

Supplement: Supplementary file 1 [file cancers-16-02629-s001.zip › File S2. Western blot/vsig8_1.tif]

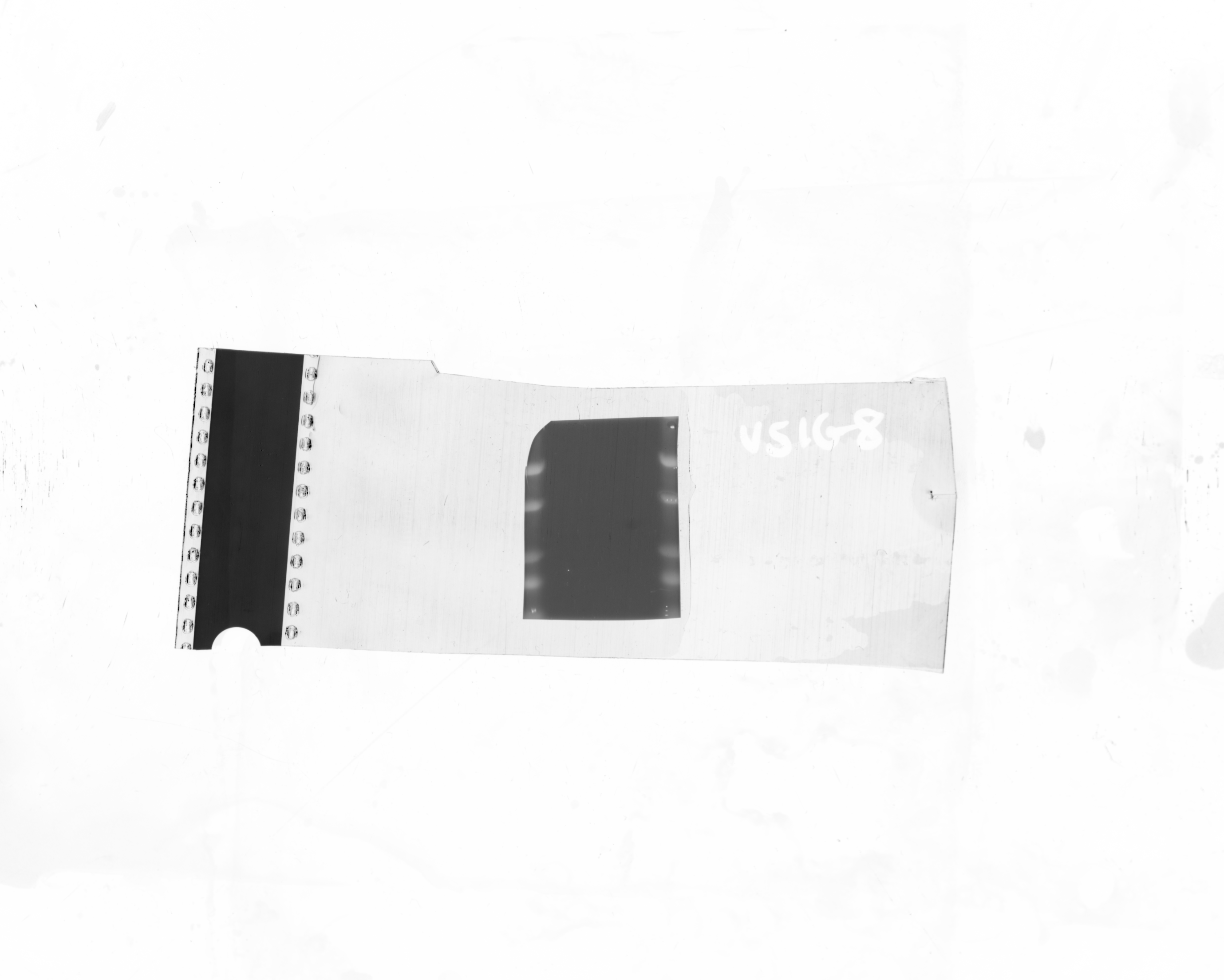

Supplement: Supplementary file 1 [file cancers-16-02629-s001.zip › File S2. Western blot/vsig8_2 ladder.tif]

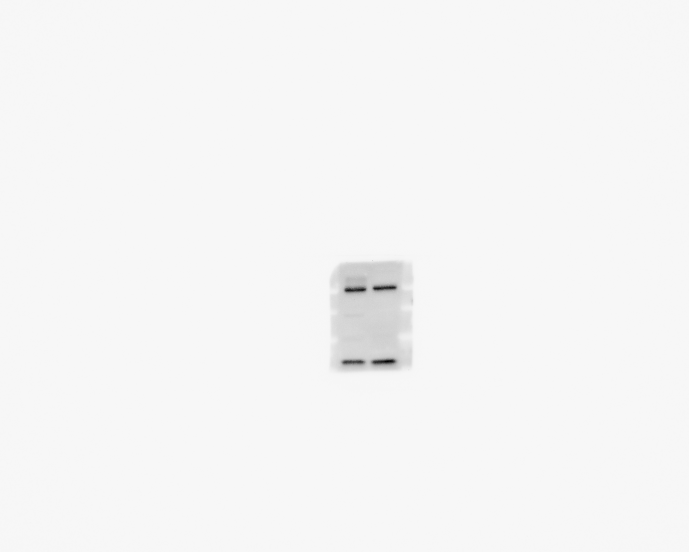

Supplement: Supplementary file 1 [file cancers-16-02629-s001.zip › File S2. Western blot/vsigthree_1.tif]

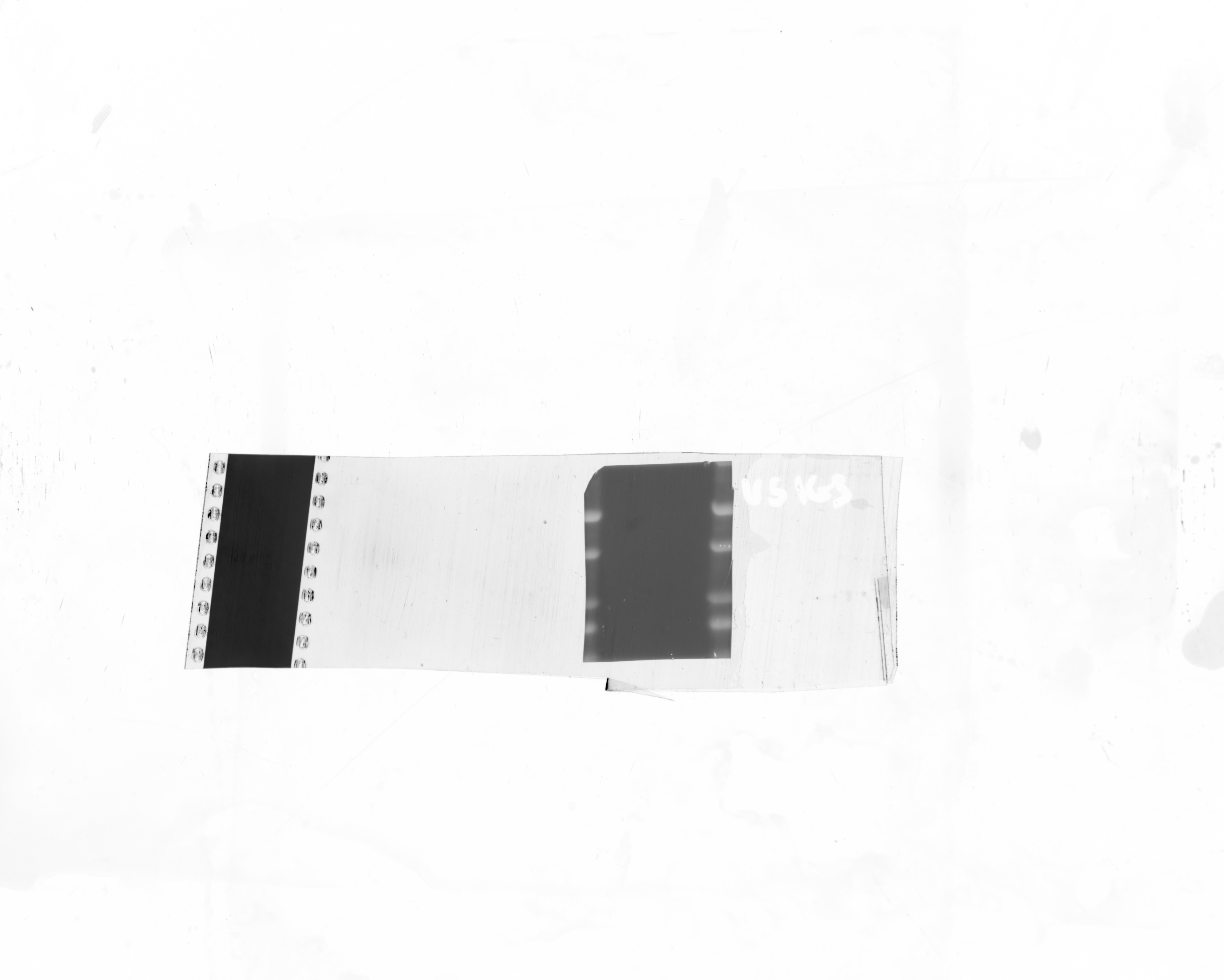

Supplement: Supplementary file 1 [file cancers-16-02629-s001.zip › File S2. Western blot/vsigthree_2 ladder.tif]
